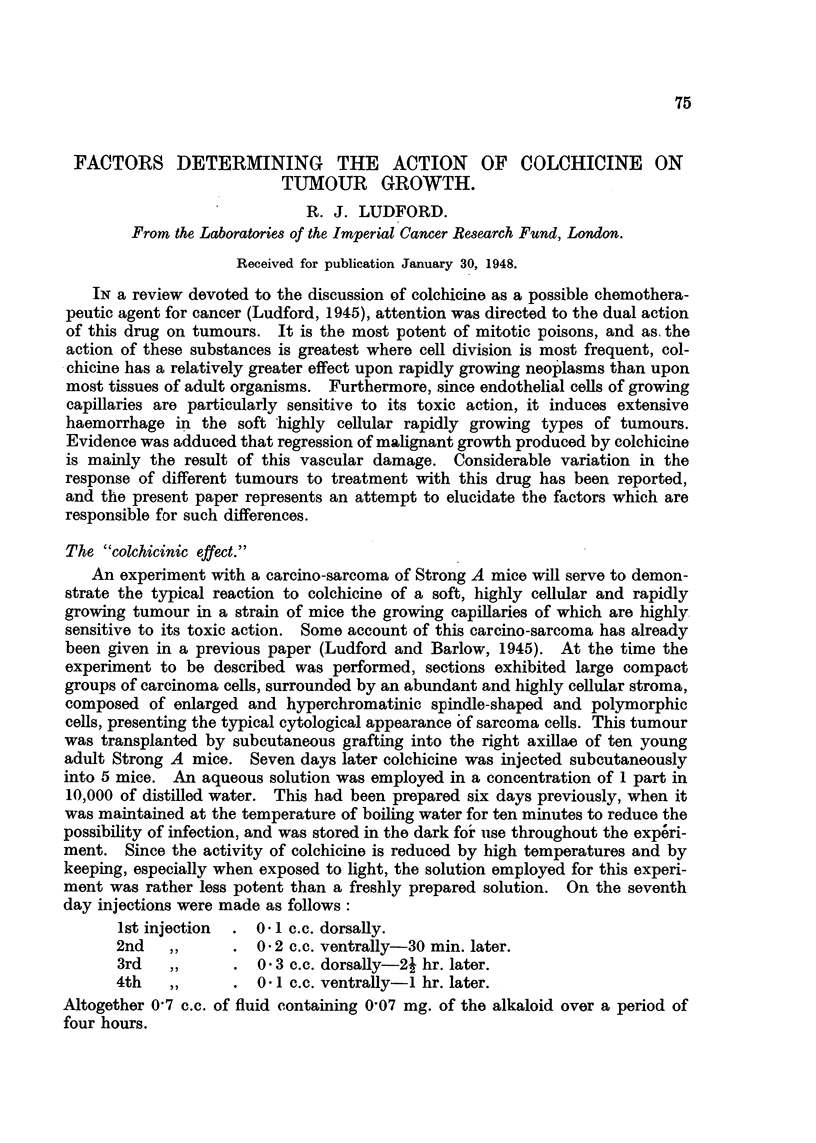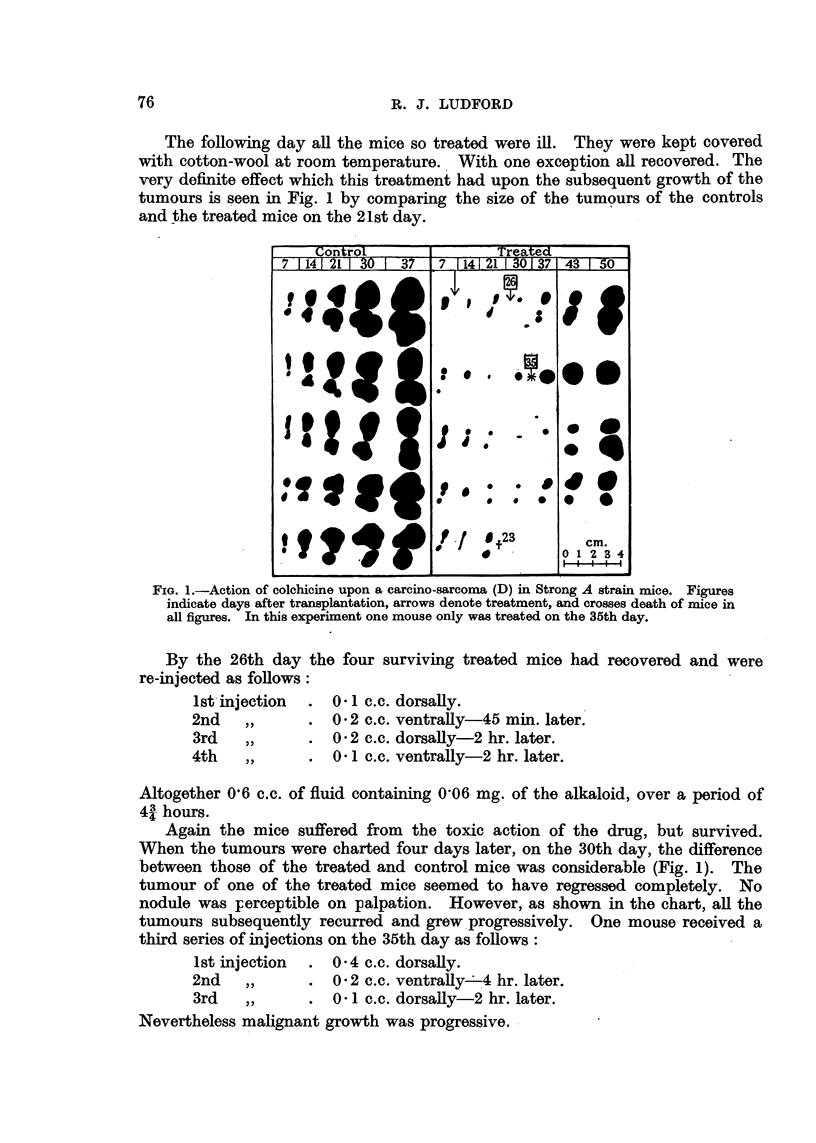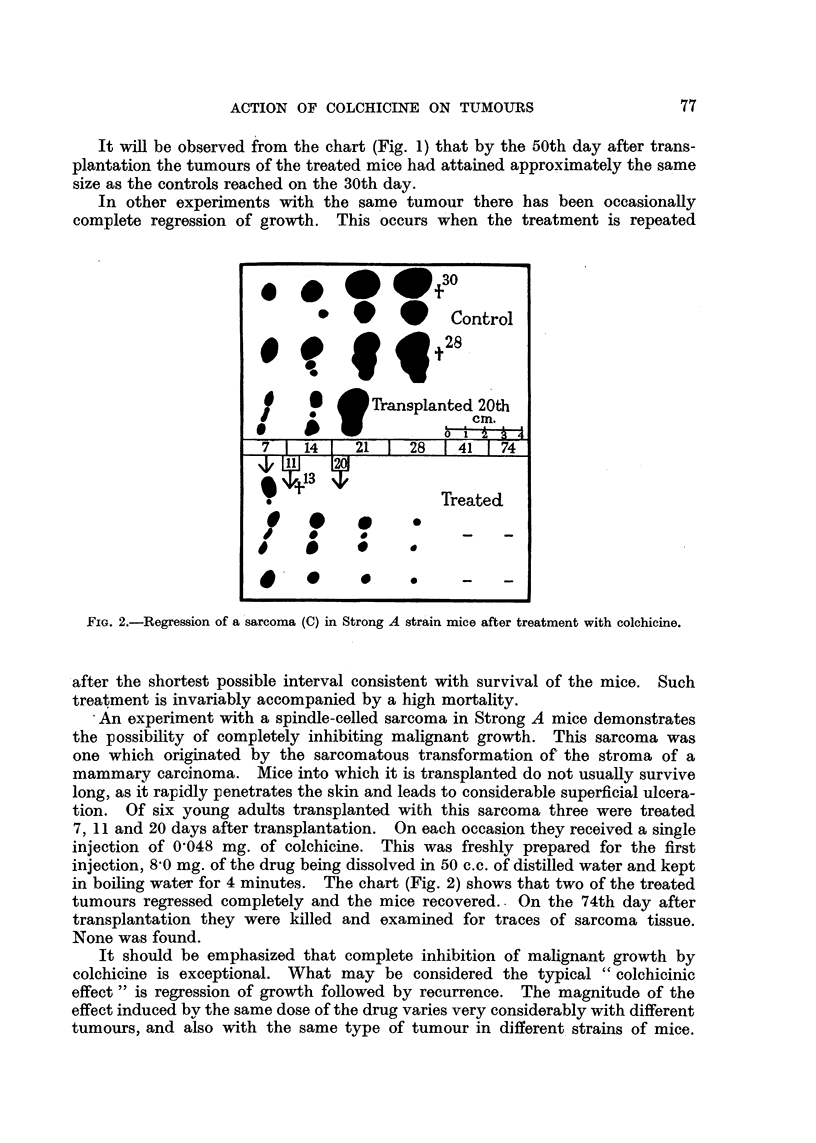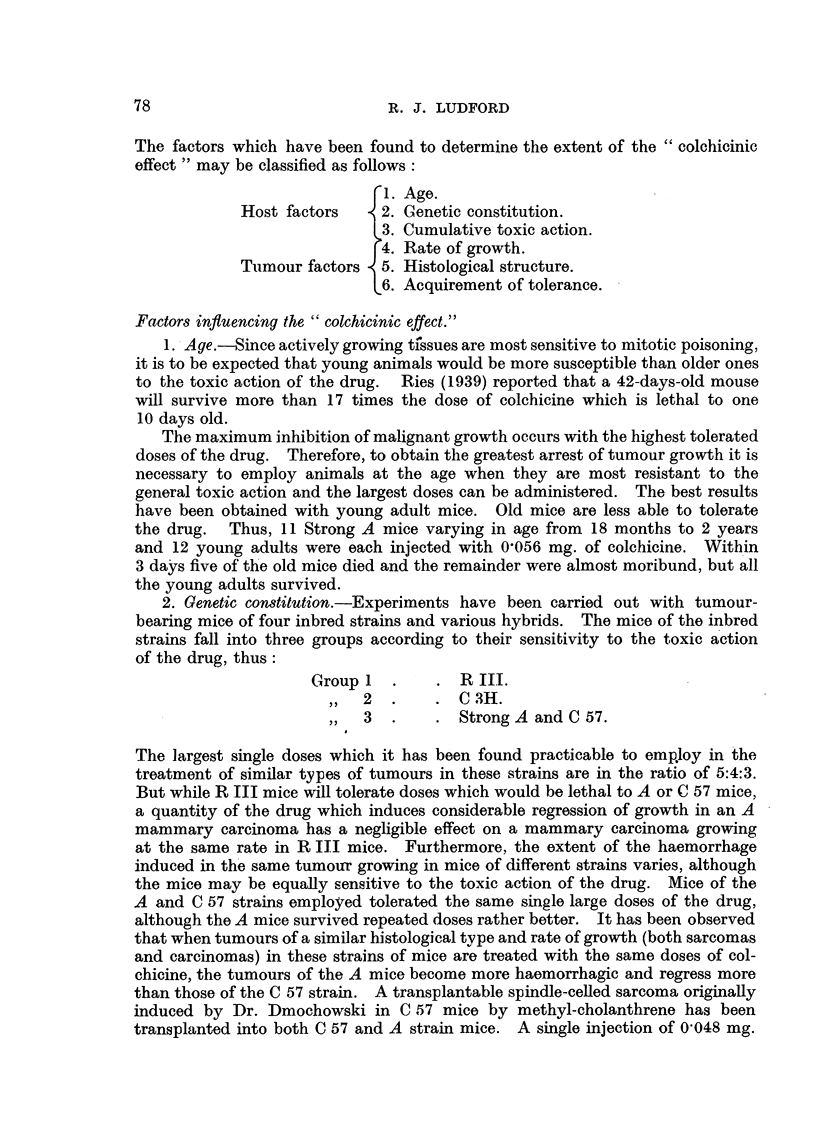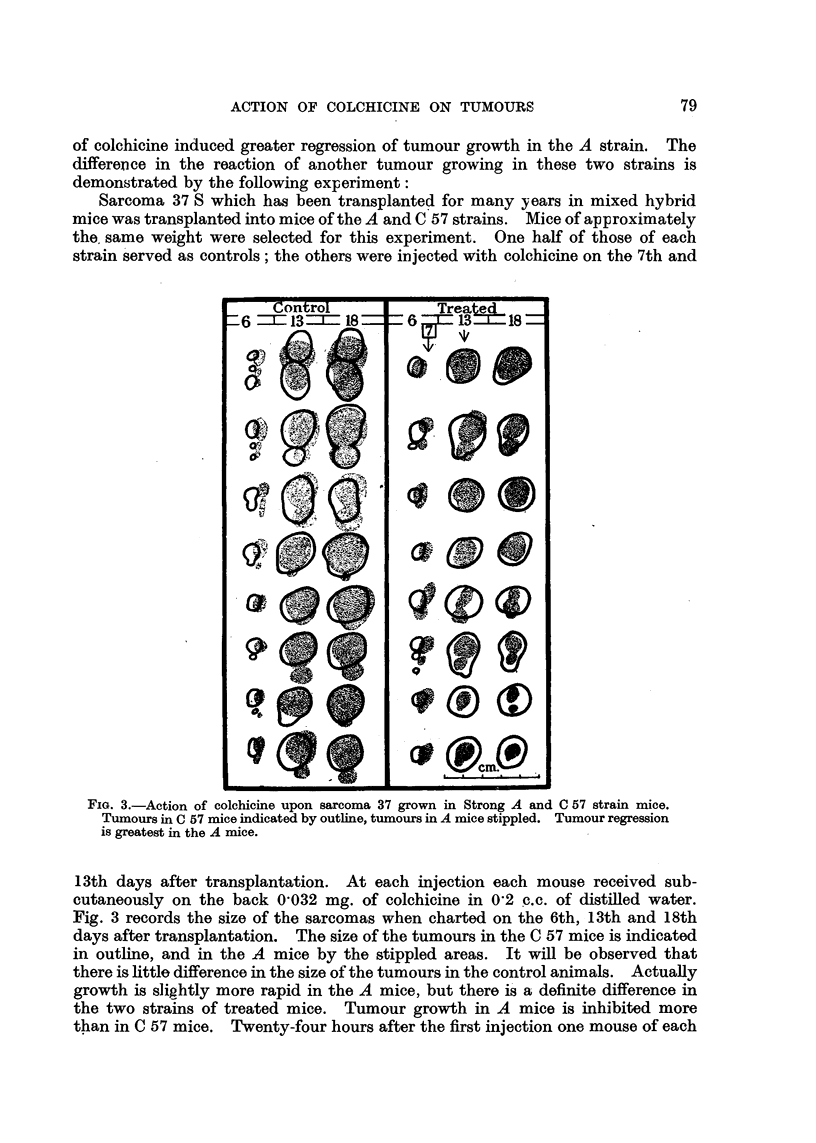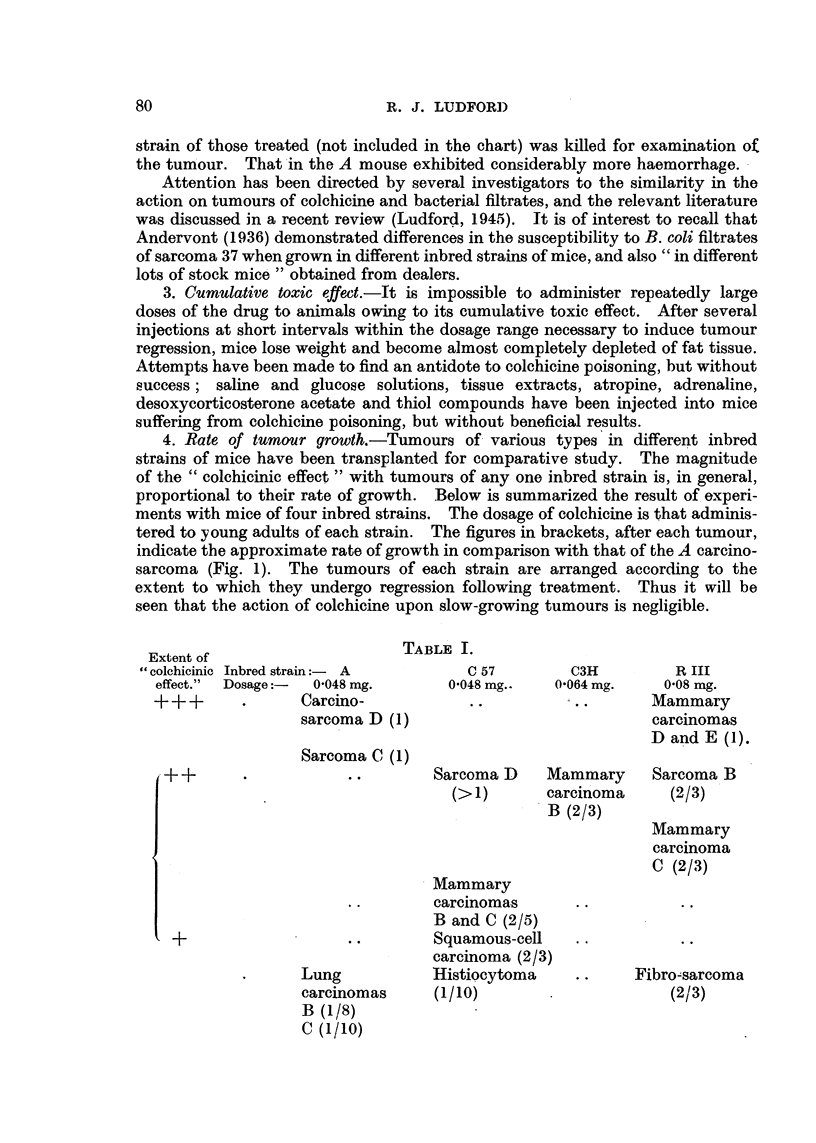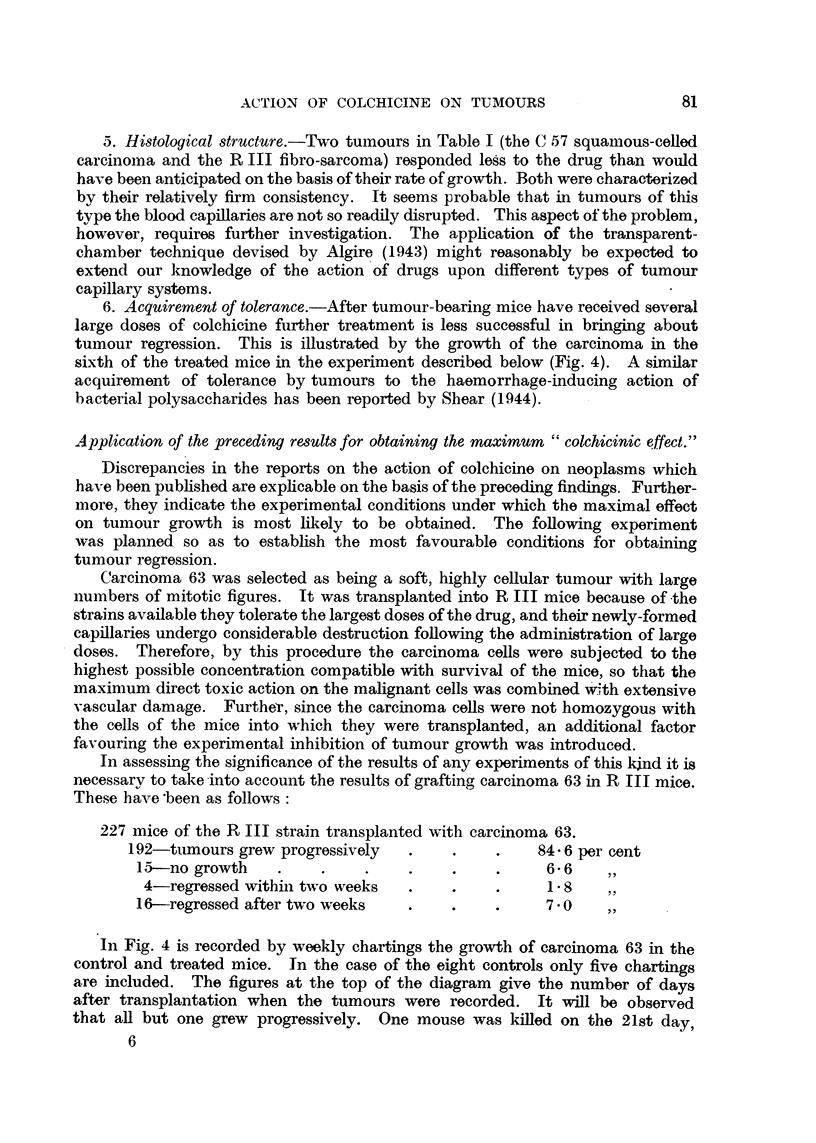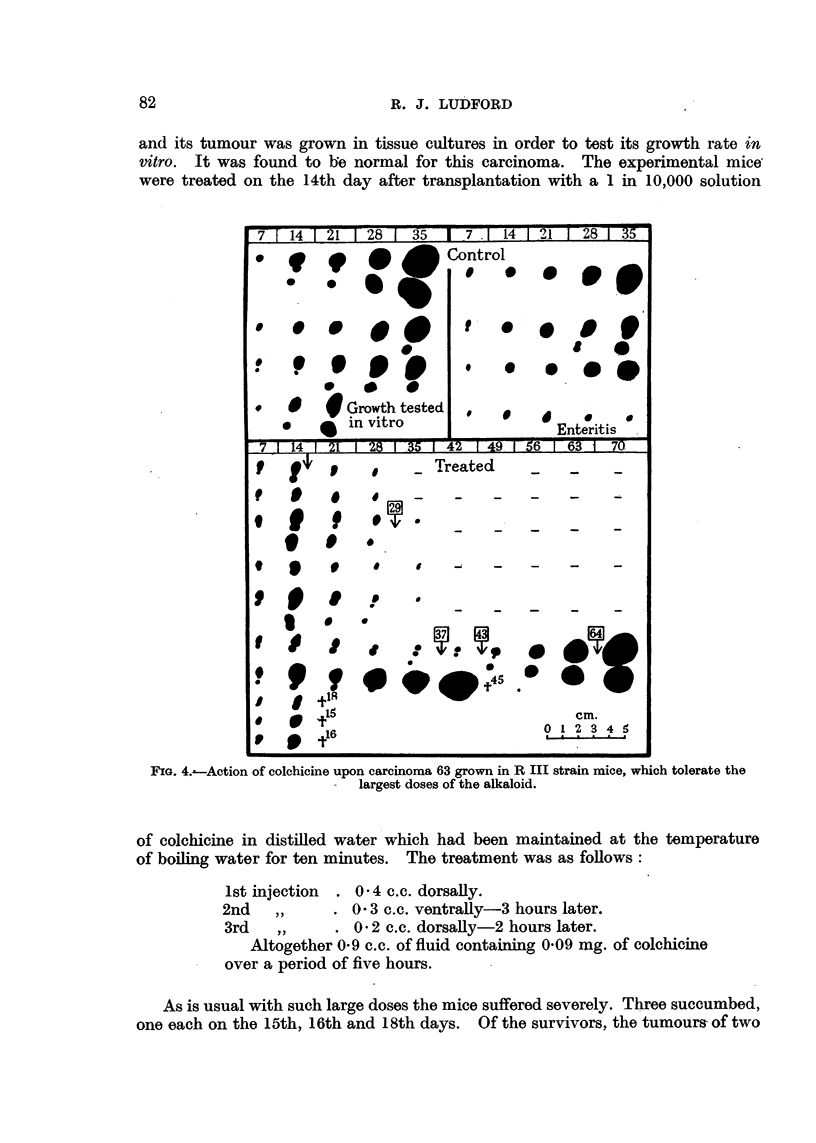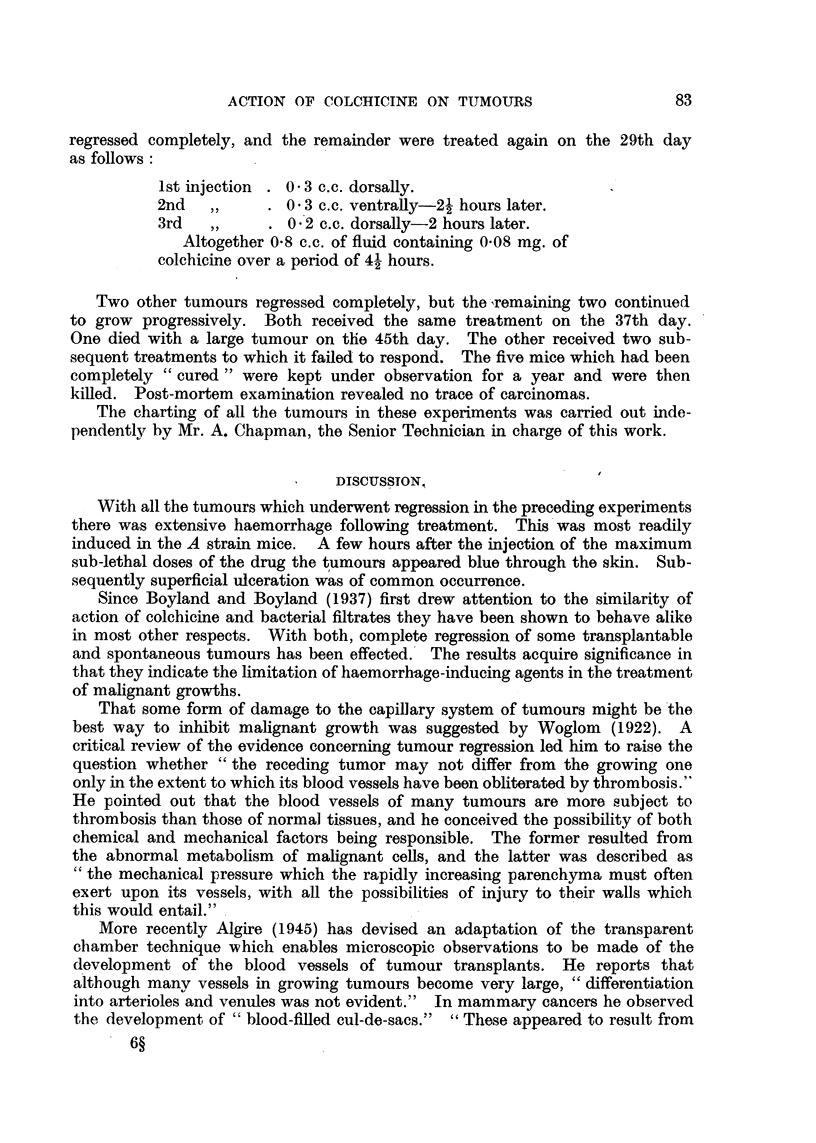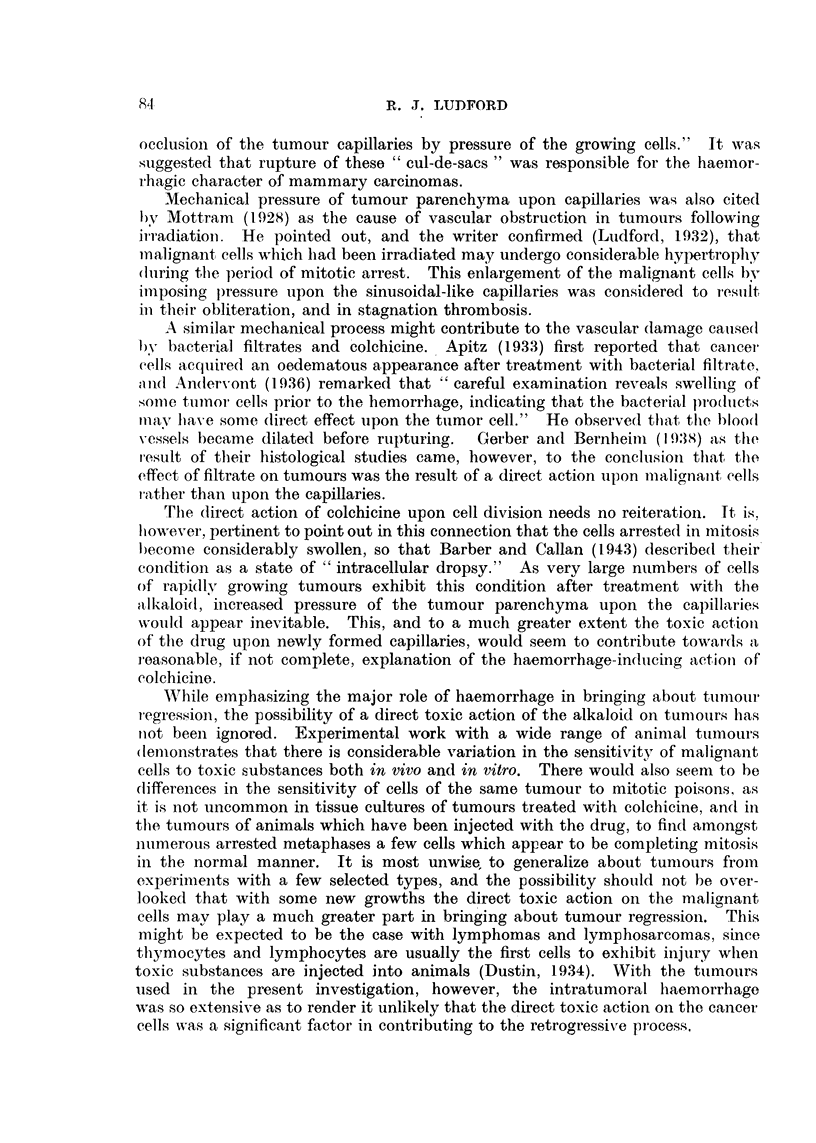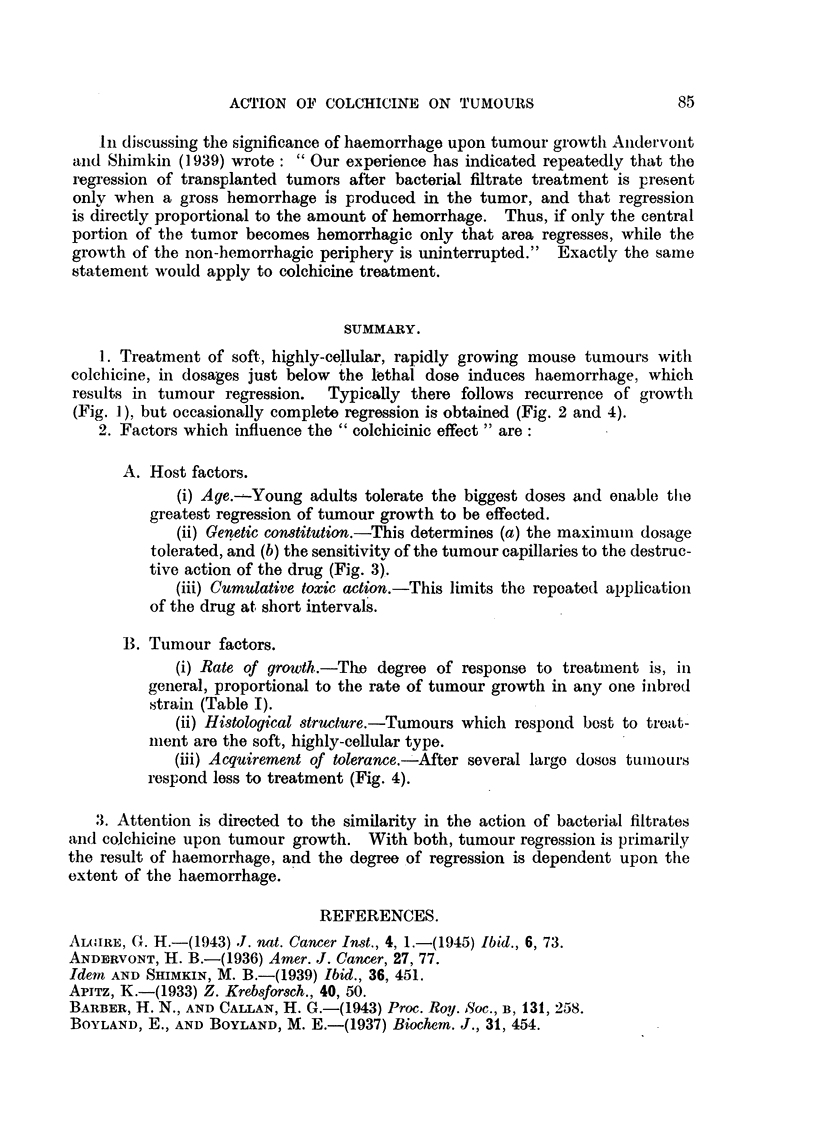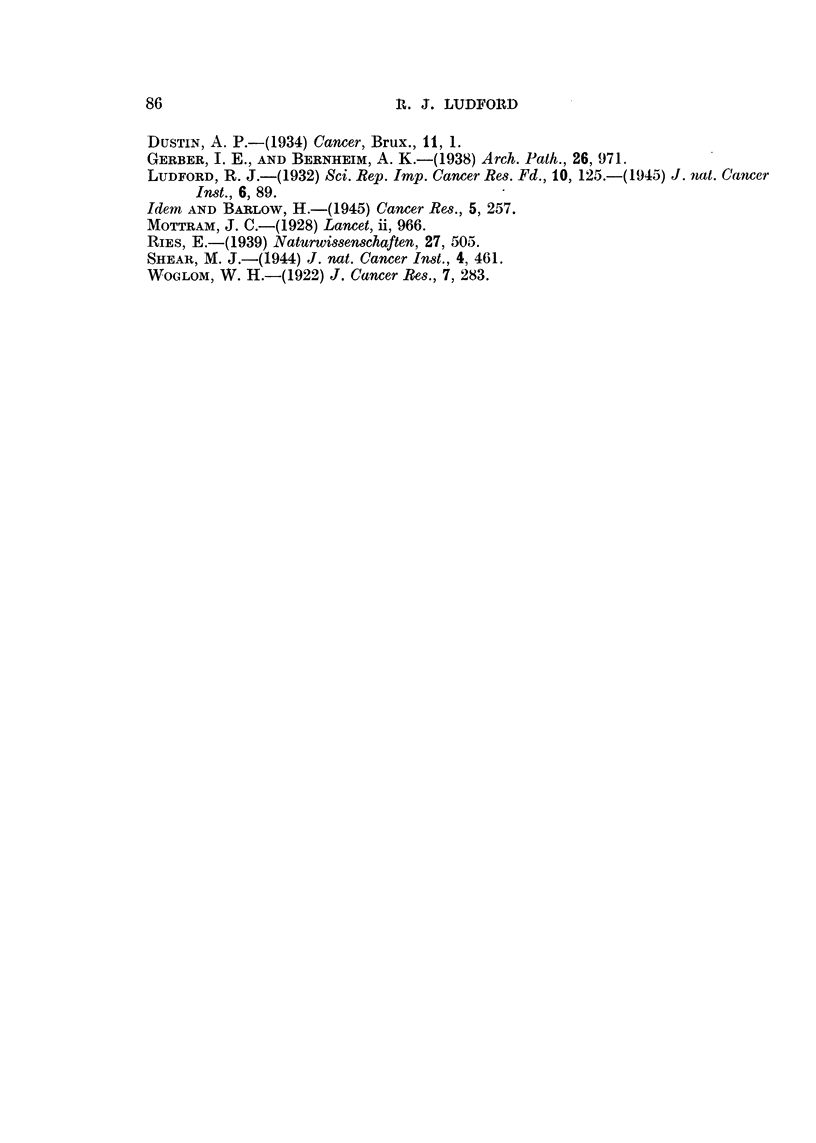# Factors Determining the Action of Colchicine on Tumour Growth

**DOI:** 10.1038/bjc.1948.10

**Published:** 1948-03

**Authors:** R. J. Ludford


### FACTORS DETERMINING THE ACTION OF COLCHICINE ON TUMOUR GROWTH.

R. J. LUDFORD.

*From the Laboratories of the Imperial Cancer Research Fund, London.*

Received for publication January 30, 1948.

IN a review devoted to the discussion of colchicine as a possible chemotherapeutic agent for cancer (Ludford, 1945), attention was directed to the dual action of this drug on tumours. It is the most potent of mitotic poisons, and as the action of these substances is greatest where cell division is most frequent, colchicine has a relatively greater effect upon rapidly growing neoplasms than upon most tissues of adult organisms. Furthermore, since endothelial cells of growing capillaries are particularly sensitive to its toxic action, it induces extensive haemorrhage in the soft highly cellular rapidly growing types of tumours. Evidence was adduced that regression of malignant growth produced by colchicine is mainly the result of this vascular damage. Considerable variation in the response of different tumours to treatment with this drug has been reported, and the present paper represents an attempt to elucidate the factors which are responsible for such differences.

*The "colchicinic effect."*

An experiment with a carcino-sarcoma of Strong *A* mice will serve to demonstrate the typical reaction to colchicine of a soft, highly cellular and rapidly growing tumour in a strain of mice the growing capillaries of which are highly sensitive to its toxic action. Some account of this carcino-sarcoma has already been given in a previous paper (Ludford and Barlow, 1945). At the time the experiment to be described was performed, sections exhibited large compact groups of carcinoma cells, surrounded by an abundant and highly cellular stroma, composed of enlarged and hyperchromatinic spindle-shaped and polymorphic cells, presenting the typical cytological appearance of sarcoma cells. This tumour was transplanted by subcutaneous grafting into the right axillae of ten young adult Strong *A* mice. Seven days later colchicine was injected subcutaneously into 5 mice. An aqueous solution was employed in a concentration of 1 part in 10,000 of distilled water. This had been prepared six days previously, when it was maintained at the temperature of boiling water for ten minutes to reduce the possibility of infection, and was stored in the dark for use throughout the experiment. Since the activity of colchicine is reduced by high temperatures and by keeping, especially when exposed to light, the solution employed for this experiment was rather less potent than a freshly prepared solution. On the seventh day injections were made as follows :

| | | |
|---|---|---|
| 1st injection | . | 0·1 c.c. dorsally. |
| 2nd ,, | . | 0·2 c.c. ventrally—30 min. later. |
| 3rd ,, | . | 0·3 c.c. dorsally—2½ hr. later. |
| 4th ,, | . | 0·1 c.c. ventrally—1 hr. later. |

Altogether 0·7 c.c. of fluid containing 0·07 mg. of the alkaloid over a period of four hours.

The following day all the mice so treated were ill. They were kept covered with cotton-wool at room temperature. With one exception all recovered. The very definite effect which this treatment had upon the subsequent growth of the tumours is seen in Fig. 1 by comparing the size of the tumours of the controls and the treated mice on the 21st day.

FIG. 1.—Action of colchicine upon a carcino-sarcoma (D) in Strong *A* strain mice. Figures indicate days after transplantation, arrows denote treatment, and crosses death of mice in all figures. In this experiment one mouse only was treated on the 35th day.

By the 26th day the four surviving treated mice had recovered and were re-injected as follows:

| | | |
|---|---|---|
| 1st injection | . | 0·1 c.c. dorsally. |
| 2nd ,, | . | 0·2 c.c. ventrally—45 min. later. |
| 3rd ,, | . | 0·2 c.c. dorsally—2 hr. later. |
| 4th ,, | . | 0·1 c.c. ventrally—2 hr. later. |

Altogether 0·6 c.c. of fluid containing 0·06 mg. of the alkaloid, over a period of $4\frac{3}{4}$ hours.

Again the mice suffered from the toxic action of the drug, but survived. When the tumours were charted four days later, on the 30th day, the difference between those of the treated and control mice was considerable (Fig. 1). The tumour of one of the treated mice seemed to have regressed completely. No nodule was perceptible on palpation. However, as shown in the chart, all the tumours subsequently recurred and grew progressively. One mouse received a third series of injections on the 35th day as follows:

| | | |
|---|---|---|
| 1st injection | . | 0·4 c.c. dorsally. |
| 2nd ,, | . | 0·2 c.c. ventrally—4 hr. later. |
| 3rd ,, | . | 0·1 c.c. dorsally—2 hr. later. |

Nevertheless malignant growth was progressive.

It will be observed from the chart (Fig. 1) that by the 50th day after transplantation the tumours of the treated mice had attained approximately the same size as the controls reached on the 30th day.

In other experiments with the same tumour there has been occasionally complete regression of growth. This occurs when the treatment is repeated

FIG. 2.—Regression of a sarcoma (C) in Strong *A* strain mice after treatment with colchicine.

after the shortest possible interval consistent with survival of the mice. Such treatment is invariably accompanied by a high mortality.

An experiment with a spindle-celled sarcoma in Strong *A* mice demonstrates the possibility of completely inhibiting malignant growth. This sarcoma was one which originated by the sarcomatous transformation of the stroma of a mammary carcinoma. Mice into which it is transplanted do not usually survive long, as it rapidly penetrates the skin and leads to considerable superficial ulceration. Of six young adults transplanted with this sarcoma three were treated 7, 11 and 20 days after transplantation. On each occasion they received a single injection of 0·048 mg. of colchicine. This was freshly prepared for the first injection, 8·0 mg. of the drug being dissolved in 50 c.c. of distilled water and kept in boiling water for 4 minutes. The chart (Fig. 2) shows that two of the treated tumours regressed completely and the mice recovered. On the 74th day after transplantation they were killed and examined for traces of sarcoma tissue. None was found.

It should be emphasized that complete inhibition of malignant growth by colchicine is exceptional. What may be considered the typical " colchicinic effect " is regression of growth followed by recurrence. The magnitude of the effect induced by the same dose of the drug varies very considerably with different tumours, and also with the same type of tumour in different strains of mice.

The factors which have been found to determine the extent of the "colchicinic effect" may be classified as follows:

| | |
|---|---|
| Host factors | 1. Age.<br>2. Genetic constitution.<br>3. Cumulative toxic action. |
| Tumour factors | 4. Rate of growth.<br>5. Histological structure.<br>6. Acquirement of tolerance. |

*Factors influencing the "colchicinic effect."*

1. *Age.*—Since actively growing tissues are most sensitive to mitotic poisoning, it is to be expected that young animals would be more susceptible than older ones to the toxic action of the drug. Ries (1939) reported that a 42-days-old mouse will survive more than 17 times the dose of colchicine which is lethal to one 10 days old.

The maximum inhibition of malignant growth occurs with the highest tolerated doses of the drug. Therefore, to obtain the greatest arrest of tumour growth it is necessary to employ animals at the age when they are most resistant to the general toxic action and the largest doses can be administered. The best results have been obtained with young adult mice. Old mice are less able to tolerate the drug. Thus, 11 Strong *A* mice varying in age from 18 months to 2 years and 12 young adults were each injected with 0·056 mg. of colchicine. Within 3 days five of the old mice died and the remainder were almost moribund, but all the young adults survived.

2. *Genetic constitution.*—Experiments have been carried out with tumour-bearing mice of four inbred strains and various hybrids. The mice of the inbred strains fall into three groups according to their sensitivity to the toxic action of the drug, thus:

| | |
|---|---|
| Group 1 . . | R III. |
| ,, 2 . . | C 3H. |
| ,, 3 . . | Strong *A* and C 57. |

The largest single doses which it has been found practicable to employ in the treatment of similar types of tumours in these strains are in the ratio of 5:4:3. But while R III mice will tolerate doses which would be lethal to *A* or C 57 mice, a quantity of the drug which induces considerable regression of growth in an *A* mammary carcinoma has a negligible effect on a mammary carcinoma growing at the same rate in R III mice. Furthermore, the extent of the haemorrhage induced in the same tumour growing in mice of different strains varies, although the mice may be equally sensitive to the toxic action of the drug. Mice of the *A* and C 57 strains employed tolerated the same single large doses of the drug, although the *A* mice survived repeated doses rather better. It has been observed that when tumours of a similar histological type and rate of growth (both sarcomas and carcinomas) in these strains of mice are treated with the same doses of colchicine, the tumours of the *A* mice become more haemorrhagic and regress more than those of the C 57 strain. A transplantable spindle-celled sarcoma originally induced by Dr. Dmochowski in C 57 mice by methyl-cholanthrene has been transplanted into both C 57 and *A* strain mice. A single injection of 0·048 mg.

of colchicine induced greater regression of tumour growth in the *A* strain. The difference in the reaction of another tumour growing in these two strains is demonstrated by the following experiment:

Sarcoma 37 S which has been transplanted for many years in mixed hybrid mice was transplanted into mice of the *A* and C 57 strains. Mice of approximately the same weight were selected for this experiment. One half of those of each strain served as controls; the others were injected with colchicine on the 7th and

FIG. 3.—Action of colchicine upon sarcoma 37 grown in Strong *A* and C 57 strain mice. Tumours in C 57 mice indicated by outline, tumours in *A* mice stippled. Tumour regression is greatest in the *A* mice.

13th days after transplantation. At each injection each mouse received subcutaneously on the back 0·032 mg. of colchicine in 0·2 c.c. of distilled water. Fig. 3 records the size of the sarcomas when charted on the 6th, 13th and 18th days after transplantation. The size of the tumours in the C 57 mice is indicated in outline, and in the *A* mice by the stippled areas. It will be observed that there is little difference in the size of the tumours in the control animals. Actually growth is slightly more rapid in the *A* mice, but there is a definite difference in the two strains of treated mice. Tumour growth in *A* mice is inhibited more than in C 57 mice. Twenty-four hours after the first injection one mouse of each strain of those treated (not included in the chart) was killed for examination of the tumour. That in the *A* mouse exhibited considerably more haemorrhage.

Attention has been directed by several investigators to the similarity in the action on tumours of colchicine and bacterial filtrates, and the relevant literature was discussed in a recent review (Ludford, 1945). It is of interest to recall that Andervont (1936) demonstrated differences in the susceptibility to *B. coli* filtrates of sarcoma 37 when grown in different inbred strains of mice, and also " in different lots of stock mice " obtained from dealers.

3. *Cumulative toxic effect.*—It is impossible to administer repeatedly large doses of the drug to animals owing to its cumulative toxic effect. After several injections at short intervals within the dosage range necessary to induce tumour regression, mice lose weight and become almost completely depleted of fat tissue. Attempts have been made to find an antidote to colchicine poisoning, but without success ; saline and glucose solutions, tissue extracts, atropine, adrenaline, desoxycorticosterone acetate and thiol compounds have been injected into mice suffering from colchicine poisoning, but without beneficial results.

4. *Rate of tumour growth.*—Tumours of various types in different inbred strains of mice have been transplanted for comparative study. The magnitude of the " colchicinic effect " with tumours of any one inbred strain is, in general, proportional to their rate of growth. Below is summarized the result of experiments with mice of four inbred strains. The dosage of colchicine is that administered to young adults of each strain. The figures in brackets, after each tumour, indicate the approximate rate of growth in comparison with that of the *A* carcinosarcoma (Fig. 1). The tumours of each strain are arranged according to the extent to which they undergo regression following treatment. Thus it will be seen that the action of colchicine upon slow-growing tumours is negligible.

TABLE I.

| Extent of " colchicinic effect." | Inbred strain :— A<br>Dosage :— 0·048 mg. | C 57<br>0·048 mg.. | C3H<br>0·064 mg. | R III<br>0·08 mg. |
|---|---|---|---|---|
| +++ | Carcino-sarcoma D (1)<br>Sarcoma C (1) | .. | .. | Mammary carcinomas D and E (1). |
| ++ | .. | Sarcoma D (>1) | Mammary carcinoma B (2/3) | Sarcoma B (2/3)<br>Mammary carcinoma C (2/3) |
| | .. | Mammary carcinomas B and C (2/5) | .. | .. |
| + | .. | Squamous-cell carcinoma (2/3) | .. | .. |
| | Lung carcinomas B (1/8) C (1/10) | Histiocytoma (1/10) | .. | Fibro-sarcoma (2/3) |

5. *Histological structure.*—Two tumours in Table I (the C 57 squamous-celled carcinoma and the R III fibro-sarcoma) responded less to the drug than would have been anticipated on the basis of their rate of growth. Both were characterized by their relatively firm consistency. It seems probable that in tumours of this type the blood capillaries are not so readily disrupted. This aspect of the problem, however, requires further investigation. The application of the transparent-chamber technique devised by Algire (1943) might reasonably be expected to extend our knowledge of the action of drugs upon different types of tumour capillary systems.

6. *Acquirement of tolerance.*—After tumour-bearing mice have received several large doses of colchicine further treatment is less successful in bringing about tumour regression. This is illustrated by the growth of the carcinoma in the sixth of the treated mice in the experiment described below (Fig. 4). A similar acquirement of tolerance by tumours to the haemorrhage-inducing action of bacterial polysaccharides has been reported by Shear (1944).

*Application of the preceding results for obtaining the maximum " colchicinic effect."*

Discrepancies in the reports on the action of colchicine on neoplasms which have been published are explicable on the basis of the preceding findings. Furthermore, they indicate the experimental conditions under which the maximal effect on tumour growth is most likely to be obtained. The following experiment was planned so as to establish the most favourable conditions for obtaining tumour regression.

Carcinoma 63 was selected as being a soft, highly cellular tumour with large numbers of mitotic figures. It was transplanted into R III mice because of the strains available they tolerate the largest doses of the drug, and their newly-formed capillaries undergo considerable destruction following the administration of large doses. Therefore, by this procedure the carcinoma cells were subjected to the highest possible concentration compatible with survival of the mice, so that the maximum direct toxic action on the malignant cells was combined with extensive vascular damage. Further, since the carcinoma cells were not homozygous with the cells of the mice into which they were transplanted, an additional factor favouring the experimental inhibition of tumour growth was introduced.

In assessing the significance of the results of any experiments of this kind it is necessary to take into account the results of grafting carcinoma 63 in R III mice. These have been as follows :

227 mice of the R III strain transplanted with carcinoma 63.

| | |
|---|---|
| 192—tumours grew progressively . . . | 84·6 per cent |
| 15—no growth . . . . . . | 6·6 ,, |
| 4—regressed within two weeks . . . | 1·8 ,, |
| 16—regressed after two weeks . . . | 7·0 ,, |

In Fig. 4 is recorded by weekly chartings the growth of carcinoma 63 in the control and treated mice. In the case of the eight controls only five chartings are included. The figures at the top of the diagram give the number of days after transplantation when the tumours were recorded. It will be observed that all but one grew progressively. One mouse was killed on the 21st day, and its tumour was grown in tissue cultures in order to test its growth rate *in vitro*. It was found to be normal for this carcinoma. The experimental mice were treated on the 14th day after transplantation with a 1 in 10,000 solution

FIG. 4.—Action of colchicine upon carcinoma 63 grown in R III strain mice, which tolerate the largest doses of the alkaloid.

of colchicine in distilled water which had been maintained at the temperature of boiling water for ten minutes. The treatment was as follows :

1st injection . 0·4 c.c. dorsally.
2nd ,, . 0·3 c.c. ventrally—3 hours later.
3rd ,, . 0·2 c.c. dorsally—2 hours later.

Altogether 0·9 c.c. of fluid containing 0·09 mg. of colchicine over a period of five hours.

As is usual with such large doses the mice suffered severely. Three succumbed, one each on the 15th, 16th and 18th days. Of the survivors, the tumours of two regressed completely, and the remainder were treated again on the 29th day as follows :

1st injection . 0·3 c.c. dorsally.
2nd ,, . 0·3 c.c. ventrally—2½ hours later.
3rd ,, . 0·2 c.c. dorsally—2 hours later.
Altogether 0·8 c.c. of fluid containing 0·08 mg. of colchicine over a period of 4½ hours.

Two other tumours regressed completely, but the remaining two continued to grow progressively. Both received the same treatment on the 37th day. One died with a large tumour on the 45th day. The other received two subsequent treatments to which it failed to respond. The five mice which had been completely " cured " were kept under observation for a year and were then killed. Post-mortem examination revealed no trace of carcinomas.

The charting of all the tumours in these experiments was carried out independently by Mr. A. Chapman, the Senior Technician in charge of this work.

### DISCUSSION.

With all the tumours which underwent regression in the preceding experiments there was extensive haemorrhage following treatment. This was most readily induced in the *A* strain mice. A few hours after the injection of the maximum sub-lethal doses of the drug the tumours appeared blue through the skin. Subsequently superficial ulceration was of common occurrence.

Since Boyland and Boyland (1937) first drew attention to the similarity of action of colchicine and bacterial filtrates they have been shown to behave alike in most other respects. With both, complete regression of some transplantable and spontaneous tumours has been effected. The results acquire significance in that they indicate the limitation of haemorrhage-inducing agents in the treatment of malignant growths.

That some form of damage to the capillary system of tumours might be the best way to inhibit malignant growth was suggested by Woglom (1922). A critical review of the evidence concerning tumour regression led him to raise the question whether " the receding tumor may not differ from the growing one only in the extent to which its blood vessels have been obliterated by thrombosis." He pointed out that the blood vessels of many tumours are more subject to thrombosis than those of normal tissues, and he conceived the possibility of both chemical and mechanical factors being responsible. The former resulted from the abnormal metabolism of malignant cells, and the latter was described as " the mechanical pressure which the rapidly increasing parenchyma must often exert upon its vessels, with all the possibilities of injury to their walls which this would entail."

More recently Algire (1945) has devised an adaptation of the transparent chamber technique which enables microscopic observations to be made of the development of the blood vessels of tumour transplants. He reports that although many vessels in growing tumours become very large, " differentiation into arterioles and venules was not evident." In mammary cancers he observed the development of " blood-filled cul-de-sacs." " These appeared to result from occlusion of the tumour capillaries by pressure of the growing cells." It was suggested that rupture of these "cul-de-sacs" was responsible for the haemorrhagic character of mammary carcinomas.

Mechanical pressure of tumour parenchyma upon capillaries was also cited by Mottram (1928) as the cause of vascular obstruction in tumours following irradiation. He pointed out, and the writer confirmed (Ludford, 1932), that malignant cells which had been irradiated may undergo considerable hypertrophy during the period of mitotic arrest. This enlargement of the malignant cells by imposing pressure upon the sinusoidal-like capillaries was considered to result in their obliteration, and in stagnation thrombosis.

A similar mechanical process might contribute to the vascular damage caused by bacterial filtrates and colchicine. Apitz (1933) first reported that cancer cells acquired an oedematous appearance after treatment with bacterial filtrate, and Andervont (1936) remarked that "careful examination reveals swelling of some tumor cells prior to the hemorrhage, indicating that the bacterial products may have some direct effect upon the tumor cell." He observed that the blood vessels became dilated before rupturing. Gerber and Bernheim (1938) as the result of their histological studies came, however, to the conclusion that the effect of filtrate on tumours was the result of a direct action upon malignant cells rather than upon the capillaries.

The direct action of colchicine upon cell division needs no reiteration. It is, however, pertinent to point out in this connection that the cells arrested in mitosis become considerably swollen, so that Barber and Callan (1943) described their condition as a state of "intracellular dropsy." As very large numbers of cells of rapidly growing tumours exhibit this condition after treatment with the alkaloid, increased pressure of the tumour parenchyma upon the capillaries would appear inevitable. This, and to a much greater extent the toxic action of the drug upon newly formed capillaries, would seem to contribute towards a reasonable, if not complete, explanation of the haemorrhage-inducing action of colchicine.

While emphasizing the major role of haemorrhage in bringing about tumour regression, the possibility of a direct toxic action of the alkaloid on tumours has not been ignored. Experimental work with a wide range of animal tumours demonstrates that there is considerable variation in the sensitivity of malignant cells to toxic substances both *in vivo* and *in vitro.* There would also seem to be differences in the sensitivity of cells of the same tumour to mitotic poisons, as it is not uncommon in tissue cultures of tumours treated with colchicine, and in the tumours of animals which have been injected with the drug, to find amongst numerous arrested metaphases a few cells which appear to be completing mitosis in the normal manner. It is most unwise to generalize about tumours from experiments with a few selected types, and the possibility should not be overlooked that with some new growths the direct toxic action on the malignant cells may play a much greater part in bringing about tumour regression. This might be expected to be the case with lymphomas and lymphosarcomas, since thymocytes and lymphocytes are usually the first cells to exhibit injury when toxic substances are injected into animals (Dustin, 1934). With the tumours used in the present investigation, however, the intratumoral haemorrhage was so extensive as to render it unlikely that the direct toxic action on the cancer cells was a significant factor in contributing to the retrogressive process.

In discussing the significance of haemorrhage upon tumour growth Andervont and Shimkin (1939) wrote : " Our experience has indicated repeatedly that the regression of transplanted tumors after bacterial filtrate treatment is present only when a gross hemorrhage is produced in the tumor, and that regression is directly proportional to the amount of hemorrhage. Thus, if only the central portion of the tumor becomes hemorrhagic only that area regresses, while the growth of the non-hemorrhagic periphery is uninterrupted." Exactly the same statement would apply to colchicine treatment.

### SUMMARY.

1. Treatment of soft, highly-cellular, rapidly growing mouse tumours with colchicine, in dosages just below the lethal dose induces haemorrhage, which results in tumour regression. Typically there follows recurrence of growth (Fig. 1), but occasionally complete regression is obtained (Fig. 2 and 4).

2. Factors which influence the " colchicinic effect " are :

A. Host factors.

(i) *Age.*—Young adults tolerate the biggest doses and enable the greatest regression of tumour growth to be effected.

(ii) *Genetic constitution.*—This determines (*a*) the maximum dosage tolerated, and (*b*) the sensitivity of the tumour capillaries to the destructive action of the drug (Fig. 3).

(iii) *Cumulative toxic action.*—This limits the repeated application of the drug at short intervals.

B. Tumour factors.

(i) *Rate of growth.*—The degree of response to treatment is, in general, proportional to the rate of tumour growth in any one inbred strain (Table I).

(ii) *Histological structure.*—Tumours which respond best to treatment are the soft, highly-cellular type.

(iii) *Acquirement of tolerance.*—After several large doses tumours respond less to treatment (Fig. 4).

3. Attention is directed to the similarity in the action of bacterial filtrates and colchicine upon tumour growth. With both, tumour regression is primarily the result of haemorrhage, and the degree of regression is dependent upon the extent of the haemorrhage.

### REFERENCES.

ALGIRE, G. H.—(1943) *J. nat. Cancer Inst.*, **4**, 1.—(1945) *Ibid.*, **6**, 73.
ANDERVONT, H. B.—(1936) *Amer. J. Cancer*, **27**, 77.
*Idem* AND SHIMKIN, M. B.—(1939) *Ibid.*, **36**, 451.
APITZ, K.—(1933) *Z. Krebsforsch.*, **40**, 50.
BARBER, H. N., AND CALLAN, H. G.—(1943) *Proc. Roy. Soc.*, B, **131**, 258.
BOYLAND, E., AND BOYLAND, M. E.—(1937) *Biochem. J.*, **31**, 454.

DUSTIN, A. P.—(1934) *Cancer*, Brux., **11**, 1.
GERBER, I. E., AND BERNHEIM, A. K.—(1938) *Arch. Path.*, **26**, 971.
LUDFORD, R. J.—(1932) *Sci. Rep. Imp. Cancer Res. Fd.*, **10**, 125.—(1945) *J. nat. Cancer Inst.*, **6**, 89.
*Idem* AND BARLOW, H.—(1945) *Cancer Res.*, **5**, 257.
MOTTRAM, J. C.—(1928) *Lancet*, ii, 966.
RIES, E.—(1939) *Naturwissenschaften*, **27**, 505.
SHEAR, M. J.—(1944) *J. nat. Cancer Inst.*, **4**, 461.
WOGLOM, W. H.—(1922) *J. Cancer Res.*, **7**, 283.